# What can be expected to be seen in a Neurology ward? Eleven-year experience in a Brazilian university hospital

**DOI:** 10.1590/0004-282X-ANP-2020-0310

**Published:** 2021-06-16

**Authors:** Daniel Gabay MOREIRA, Enedina Maria Lobato de OLIVEIRA, Fernando Morgadinho dos Santos COELHO, Henrique Ballalai FERRAZ, Silvio FRANCISCO, Vanderci BORGES, Denis Bernardi BICHUETTI

**Affiliations:** 1 Universidade Federal de São Paulo, Escola Paulista de Medicina, Disciplina de Neurologia, São Paulo SP, Brazil. Universidade Federal de São Paulo Universidade Federal de São Paulo Escola Paulista de Medicina Disciplina de Neurologia São Paulo SP Brazil

**Keywords:** Neurology, Patients' Rooms, Prevalence, Disease, Neurologia, Enfermaria, Prevalência, Doença

## Abstract

**Background:**

Neurological disorders are significant causes of morbidity and mortality worldwide. However, data about general neurological inpatient admissions in Brazil is limited. **Objective:** To investigate the prevalence of neurological disorders according to disease group and lesion site among patients admitted to a general Neurology ward.

**Methods:**

This was an observational and descriptive study. The hospital discharge database for the Neurology ward was surveyed in accordance with the International Classification of Diseases, 10^th^ edition (ICD-10), from September 2008 to October 2019. The final diagnosis was classified into neurological disorder groups and site.

**Results:**

Overall, 2,606 clinical neurological patient files were included, with mean length of hospitalization of 16.7 days and a total of 325 readmissions (12.5%). The overall mortality rate in the ward was 3.8% (100 patients). Among all the diagnoses, cerebrovascular disease was the most prevalent (45.8%), followed by inflammatory disorders (22.2%). The brain was the most common lesion site (66.0%), followed by peripheral nerves (10.0%) and meninges and cerebrospinal fluid (7.7%).

**Conclusions:**

The disease pattern upon admission showed that a majority of the cases consisted of cerebrovascular disorders and that the brain was the most frequently affected structure, although we observed that a wide variety of cases were admitted, encompassing all neurological disorders.

## RESUMO

**Introdução:**

As doenças neurológicas representam importante causa de morbidade e mortalidade globalmente, mas informações acerca de internações hospitalares em neurologia no Brasil são limitadas.

**Objetivo:**

Investigar a prevalência de admissões neurológicas por grupo de doenças e pela topografia atendidas em uma enfermaria de neurologia geral. **Métodos:** Estudo observacional e descritivo. Avaliados diagnósticos de saída de acordo com o Código Internacional de Doenças-10 (CID-10) no período de setembro de 2008 a outubro de 2019. Os diagnósticos foram classificados em grupos de doença e por topografia.

**Resultados:**

Foram incluídos 2,606 pacientes, com tempo médio de internação de 16,7 dias e um total de 325 (12,5%) readmissões. A mortalidade geral na enfermaria foi de 100 (3,8%) pacientes. A doença cerebrovascular foi mais prevalente (45,8%), seguida das doenças inflamatórias (22,2%). A topografia encefálica foi a mais comum (66,0%), seguida de nervos periféricos (10,0%), meninges e líquido cefalorraquidiano (7,7%).

**Conclusões:**

O perfil de doenças observado neste estudo demonstrou maior prevalência das doenças cerebrovasculares e da topografia encefálica, embora uma grande variedade de doenças tenha sido admitida dentro do espectro de doenças neurológicas.

## INTRODUCTION

Neurological disorders account for nearly 12% of total deaths globally and are the main cause of overall disease burden, which is represented by the number of years of healthy life lost as the result of disability. The World Health Organization (WHO) has estimated that morbidity due to neurological diseases has overtaken diseases consequent to HIV/AIDS and malignant neoplasm^1^. In the United Kingdom National Health Service, one in every six people has a neurological condition and deaths due to neurological causes increased by 39% between 2001 and 2014, while all other causes of death decreased by 6% over the same period^2^. It has been is estimated that this impact is even greater in developing countries than in higher-income nations^3^. In Brazil, data relating to the distribution of neurological disorders is scant. Most of the studies investigating this have drawn a profile of these diseases in outpatient care settings^4^^,^^5^^,^^6^.

Hospital São Paulo is a major tertiary care hospital located in the southern district of São Paulo, a megalopolis with a population greater than 21 million inhabitants^7^. It is the major teaching hospital for Escola Paulista de Medicina, the medical school of the Universidade Federal de São Paulo. The Neurology ward of Hospital São Paulo is responsible for medical assistance for all neurological admissions from the Neurology and Neurosurgery Department, one of most traditional centers for Neurology training across the country. This unit has 18 beds: eight dedicated to clinical Neurology and ten to Neurosurgery. A multidisciplinary team that includes residents from Neurology, Nursing, Physiotherapy, Psychology and Speech and Language Pathology, all of them supervised by skilled tutors, is responsible for the medical assistance^8^.

To better understand the prevalence of major inpatient neurological disorders that are being cared for in the scenario of the 21^st^ century, we designed a retrospective study with prospectively acquired data on all admissions to the general Neurology ward over a period of 11 continuous years.

## METHODS

This was a retrospective, descriptive and observational study that used prospectively acquired data. It evaluated all patients admitted to the Neurology ward of Hospital São Paulo, which is part of the public healthcare system of Brazil, over the period from September 2008 to October 2019. The study was approved by the ethics committee of the Federal University of São Paulo. The patients’ medical records were obtained from the hospital system database, which collects information at the time of patient discharge. Thus, the study was exempted from obtaining individual consent through a statement.

We reviewed all 6,717 entries from patients admitted from 2008 to 2019. The patients were assembled in a database of diagnoses that was created in accordance with the International Classification of Diseases, 10^th^ edition (ICD-10), upon medical discharge. Since the hospital system combines clinical and surgical patients, we excluded from the analysis all non-clinical neurological patients, i.e. cases of nervous system tumors, traumatic brain and spine injury, Parkinson disease admitted for implantation of deep brain stimulation, central vascular malformations (such as intracranial aneurysm and arteriovenous malformation) and, hydrocephalus. Also, any records with incomplete data were excluded. Only patients older than 12 years were included, since this is an adult-only ward.

The following variables were collected: age, sex, length of stay, readmission, clinical outcome (death or discharge) and ICD-10 diagnosis. We subsequently classified the ICD-10 diagnoses into neurological disease groups and lesion sites, in order to understand whether residents and students were encountering different diseases, with an opportunity to evaluate patients within the entire spectrum of neurological symptoms, thus fulfilling the academic purpose of the unit. The neurological diseases were grouped as degenerative, metabolic, seizures, infectious, inflammatory, cerebrovascular and unclassified. The lesion site groups were the brain (comprising brain, midbrain and cerebellar lesions), cerebrospinal fluid, spine, peripheral nerves, neuromuscular junction or muscle, multiple site and unclassified.

The analysis was performed using Microsoft Excel® and Epi-Info^TM^ 7. Quantitative variables were presented as means and standard deviations, while qualitative variables were presented as absolute numbers and percentages.

## RESULTS

**Figure 1.**
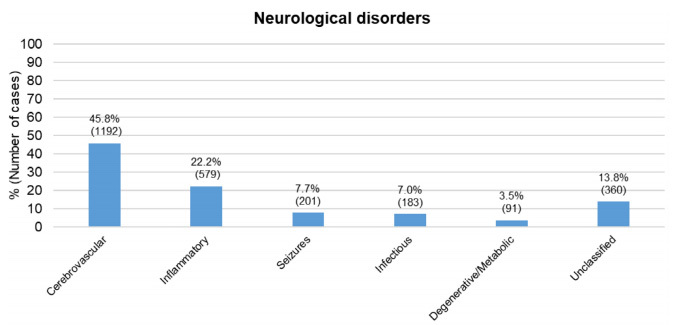
Groups of neurological disorders admitted to the Neurology ward (n=2,606).

Overall, 2,606 patients with clinical neurological diagnoses were included for analysis. Table 1 describes the demographic and general characteristics of the admissions included during the study period. The largest group was of cerebrovascular diseases (45.8%), followed by inflammatory (22.2%) and unclassified (13.8%) (Figure 1) (Table 2).The group with unclassified diagnoses encompassed patients discharged due to headache and single nerve impairments. The rest of the groups had each a percentage incidence inferior to 10%.

**Table 1.** Demographics of the 2,606 admissions over an 11-year period.

	n	%
Age (n=2,606)		
Mean±SD	48.8±18.0	-
12-20	136	5.2
21-30	361	13.8
31-40	431	16.5
41-50	393	15.1
51-60	521	20.0
61-70	438	16.8
71-80	241	9.3
81-90	75	2.9
91-99	10	0.4
Sex (n=2,606)		
Female	1,361	52.2
Male	1,245	47.8
Length of stay (days)		
Mean	16.6	-
Admissions (n=2,606)		
1^st^ admission	2,281	87.5
Readmission	325	12.5
Clinical outcome (n=2,606)		
Discharge	2,506	96.2
Death	100	3.8
Total	2,606	100.0

**Figure 2.**
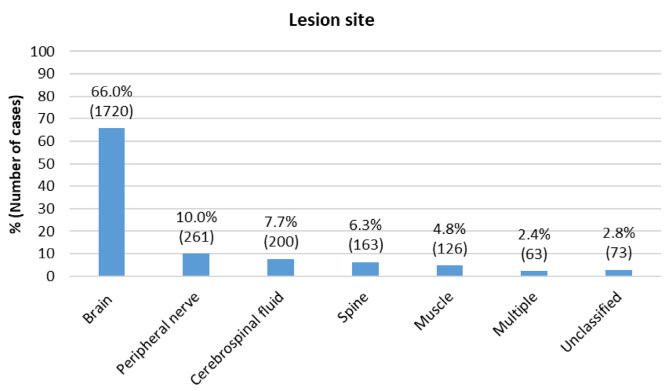
Groups according to lesion sites, admitted to the Neurology ward (n=2,606).

The most common lesion site was the brain (66.00%), followed by peripheral nerves (10.0%), meninges and cerebrospinal fluid (7.7%) and spinal cord (6.6%) (Figure 2) (Table 2). The other anatomical lesion sites each had proportions smaller than 5%. It is important to note that the multiple site group only included diagnoses of encephalomyelitis. The unclassified group represented general ICD-10 codes, for situations in which it was not possible to be certain of the correct lesion site, such as when the discharge diagnosis was filled out as “unspecified demyelinating disease of the central nervous system” or “other degenerative specified diseases of the nervous system”.

**Table 2.** Groups according to neurological disorders and lesion site admitted to the neurology ward.

	n	%
Neurological disorders (n=2,606)		
Cerebrovascular	1192	45.7
Inflammatory	579	22.2
Seizures	201	7.7
Infectious	183	7.0
Degenerative/metabolic	91	3.5
Unclassified	360	13.8
Lesion site (n=2,606)		
Brain	1720	66.0
Peripheral nerve	261	10.0
Meninges and cerebrospinal fluid	200	7.7
Spine	163	6.3
Muscle	126	4.8
Multiple sites	63	2.4
Unclassified	73	2.8
Total	2,606	100.0

## DISCUSSION

Inpatient care is clinically challenging, demands multidisciplinary coordinated care and is potentially high-priced, especially in academic hospitals^9^. Comprehending a unit’s profile of patient admissions and disease prevalence is a first step towards organizing this care and shaping educational programs in university hospitals.

Cerebrovascular diseases are the largest cause of neurological admissions worldwide, with frequencies ranging from 47.5 to 62%. Infectious diseases and seizures alternate in the second and third position, ranging from 3.9 to 21.8% and 4.8 to 9.8%, respectively^10^^,^^11^^,^^12^^,^^13^. In one of the few Brazilian inpatient series available, the cerebrovascular group was the most prevalent, with a frequency of 51%^14^. In our ward too, cerebrovascular diseases were the most prevalent group, followed by inflammatory diseases, mainly composed of multiple sclerosis, neuromyelitis optica, myasthenia gravis, Guillain-Barré syndrome and optic neuritis alone. This might be explained by the fact that Hospital São Paulo has specialized Neurology outpatient clinics that care for demyelinating and neuromuscular disease. Besides being the hospital’s main provider of acute care, our emergency unit is also an open walk-in clinic, so many patients with inflammatory diseases without diagnoses come to our hospital seeking care.

The outpatient scenario in Brazil differs from the hospitalization profile, given that the most common complaints or diseases seen in neurological consultation offices are headache, seizures, cerebrovascular diseases and dementia syndrome^4^^,^^5^. We categorized headache diagnoses in the unclassified group, as they most likely represent a symptom of inpatients’ final diagnoses, rather than a separate disease. Nonetheless, headache was included in the third most prevalent group among neurological admissions.

The diagnostic process varies among diseases and distinct specialties. There are three major spectra: clinical-dominant, laboratory-dominant and neuroimaging-dominant^15^. The role of neuroanatomy in understanding and determining possible lesion sites has long been established within the approached to neurological disorders. This is vital for guiding investigation and neurological rationale, especially in academic centers^16^. In our series, the brain was the site predominantly affected, which was not surprising considering that nearly half of our admissions were due to cerebrovascular disease, followed by inflammatory diseases mainly encompassing relapses of multiple sclerosis and central nervous system demyelinating and inflammatory diseases. Nevertheless, we found that all areas of Neurology and neurological clinical sites were being seen, which was in keeping with the purpose of an academic unit.

This study was not intended to be a complete review of clinical admissions or treatment results, which would require a complete chart review of all admitted patients. The fact that we excluded all possible surgical patients might have reduced the number of patients cared for by clinical Neurology residents that we evaluated, given that some neurosurgical patients might first have been admitted to clinical care and investigation, and were then moved to surgical care. Moreover, this study embraces a common limitation of database studies, i.e. situations of incomplete or incorrect input of data. To surmount this, a complete review of all medical records would need to be conducted, which was beyond the objectives of this report. Furthermore, it is worth mentioning that patients seeking care in Hospital São Paulo are first assisted in the neurological emergency service and many receive their complete medical treatment there and are not admitted to the ward. This biases the inpatient population towards those with more complex diseases and longer stays.

In conclusion, in this Brazilian general Neurology ward, lesions to the brain and cerebrovascular diseases accounted for the major causes of neurological admissions. The present findings contribute to a better understanding of hospitalized neurological patients and can help in future planning of allocation of effort and medical assistance priorities, as well as guiding academic centers in organizing their rotations to cover the full spectrum of neurological care.
